# Compound* Phyllanthus urinaria* L Inhibits HBV-Related HCC through HBx-SHH Pathway Axis Inactivation

**DOI:** 10.1155/2019/1635837

**Published:** 2019-03-24

**Authors:** Yun Li, Mingjie Jiang, Mingshun Li, Yingjie Chen, Chunshan Wei, Lisheng Peng, Xinliang Liu, Zhen Liu, Guangdong Tong, Daqiao Zhou, Jinsong He

**Affiliations:** Shenzhen Traditional Chinese Medicine Hospital, The Fourth Clinical Medical College of Guangzhou University of Chinese Medicine, China

## Abstract

*Compound Phyllanthus urinaria L *(CP) is a traditional formula widely used in clinical practice for hepatocellular carcinoma (HCC), especially HBV-related HCC. HBx, HBV X gene encoded X protein, has positive correlation with the abnormal SHH pathway in HBV-related HCC. So, we predicted that CP has the capability of anti-HBV-related HCC maybe via inactivating the HBx-Hedgehog pathway axis. HepG2-HBx cells, HBx overexpression, were treated with CP (70*μ*g/ml and 35 *μ*g/ml, respectively) for 48 hours and the mice which received the HepG2-HBx cells were treated with CP (625mg/kg and 300 mg/kg, respectively) for 17 days to evaluate the effect of CP on HBV-related HCC. HBx could accelerate HepG2 cells proliferation, clone formation, and migration in vitro and also could strengthen tumor growth in mice. However, CP could significantly decrease HepG2-HBx cells proliferation, clone formation, and migration in vitro and also could inhibit tumors growth in mice in a dose-dependent manner. Mechanism studies suggested that HBx upregulated the mRNA and proteins expression of Sonic hedgehog (SHH), transmembrane receptor patched (PTCH-1), smoothened (SMO), oncogene homolog transcription factors-1 (GLI-1), and oncogene homolog transcription factors-2 (GLI-2), which are compositions of the SHH pathway. CP could inhibit the mRNA and proteins expression of SHH, PTCH-1, GLI-1, and HBx. It may be one of the underlying mechanisms of CP to delay the HBV-related HCC development through the HBx-SHH pathway axis inactivation.

## 1. Introduction

Hepatocellular carcinoma (HCC) is one of the common cancers with high mortality and morbidity rate, as well as recurrence rate in spectrum of diseases. Chronic Hepatitis B virus (HBV) infection is a main risk factor for HCC development [1]. There were estimated nearly two hundred million people with HBV, which leaded to nearly one million people death all over the world in 2015[2].

HBV X gene encode X protein (HBx) is the core protein in HBV replication progression, strongly associated with tumorigenesis [3]. Growing evidence suggests that HBx protein promotes cell cycle progression, inactivates the expression of tumor suppressor gene, such as p53, and inhibits negative growth regulator [4]. However, the whole spectrum of HBx-modulating HBV-related HCC remains not fully uncovered.

The Hedgehog pathway plays a critical role in tissue repair and regeneration, as well as involved in tumorigenesis [5]. The Hedgehog pathway includes three types of Hh ligands, SHH, Indian Hedgehog (IHH), Desert Hedgehog (DHH), and one receptor patched (Ptc), which in human is PTCH-1. In the absence of ligand, GLI is inactivated and that maintains normal homeostasis. Once ligand binds to Ptc, GLI is activated and migrates to cell nucleus; a wide tissue-specific target genes are activated, such as cyclins and bcl-2, as well as its compositions GLI-1 and GLI-2 [6, 7]. Accumulating evidence from in vitro and in vivo indicated that abnormal SHH pathway activation has been found in breast cancer, prostatic cancer, and ovarian cancer as well as liver cancer [8–10]. The SHH pathway is becoming a potential target for treating cancers. In our previous study, we found that SHH, PTCH-1, and GLI-2 were overexpression in the paracarcinoma tissue of HBV-related HCC patients, and they have positive correlation with HBx (data not show), which revealed that maybe HBx activate the SHH pathway, contributing to HBV-related HCC development.

*Compound Phyllanthus urinaria L (*CP*) [Phyllanthus urinaria L, Astragalus membranaceus (Fisch.) Bunge, Scutellaria barbata D. Don, Curcuma zedoaria (Christm.) Rosc, and Cremastra appendiculata (D. Don) Makino] *is a formula widely used to treat HCC, especially HBV-related HCC in clinical practice [11]. CP has various pharmacological functions, such as antivirus and antifibrosis, and also can stimulate immune response. Except for HCC, CP also can be used to treat liver injury, hepatic fibrosis, and cirrhosis [12, 13]. However, the molecular mechanism of CP anti-HBV-related HCC is uncertain. In our previous studies, we have proved that CP has a capability of reducing the expression of HBx [14]; moreover, we also found that HBx has a positive correlation with the SHH pathway activation in HBV-related HCC patients' liver samples (data not shown). Therefore, we predicted that HBx might activate the SHH pathway to promote HBV-related HCC development, and the capability of CP anti-HBV-related HCC might be based on HBx-SHH pathway axis inactivation.

## 2. Materials and Methods

### 2.1. Preparation and HPLC Analysis of* CP*

CP* [Phyllanthus urinaria L (30g), Astragalus membranaceus (Fisch) Bunge (15g), Scutellaria barbata D. Don (10g), Curcuma zedoaria (Christm) Rosc (10g), and Cremastra appendiculata (D. Don) Makino (10g)]* purchased from the first affiliated hospital of Guangzhou university of Chinese medicine (Guangzhou, China), was soaked in 1L water for 40 min at room temperature and incubated at 100°C for 1 h, and then, the extract was concentrated at rates of 50rmp, 50°C by Rotary Evaporator (RE-2000, Yarong), and filtrated with 0.22*μ*m filter in Clean Bence (Healforce, China).

The main ingredients of CP were analyzed using an Agilent HPLC system (1260 series, Agilent Technology, Santa Clara, CA, USA) with an phenomenex Luna 5u C18(2) 100A column (250mm×4.6mm, 5 *μ*m). Signals were detected at 270 nm using a UV monitor (1260 series, Photo-Diode Array UV/V, Agilent Technology). The gallic acid, quercetin, and luteolin were quantitated using a calibration curve established by each standard (500*μ*g/ml, 502.17*μ*g/ml, and 505 *μ*g/ml, respectively) in to HPLC system. Gallic acid, quercetin, and luteolin for HPLC analysis were purchased from Yuanye Phytochemicals Ltd. (Shanghai, China).

### 2.2. Establishment of Stable Cell Lines

Lentiviral particles expressing HBx or control sequence (NC) were constructed by Wenqi Bioscience Inc. (Guangzhou, China). HepG2 cells (American Type Culture Collection) were infected with lentiviral particles expressing control sequence or HBx and then selected with 1.6ug/ml puromycin (MedChemExpress, China). After antibiotic election for around 7 days, remaining cells (HepG2-HBx; HepG2-NC) were collected and overexpression of HepG2-HBx cells was used for next studies.

### 2.3. MTT Assays

HepG2-HBx cells were cultured in DMEM supplemented with 10% fetal bovine serum (Gibco, USA) and 1% penicillin/streptomycin (Gibco, USA). HepG2-HBx cells were treated with CP at different concentrations for 24 h and 48 h; the OD_490_ and cell viability were measured with the MTT (Beyotime, Guangzhou) using Microplate reader (Thermo. USA) [15]. IC_50_ of CP was analyzed by Graphpad prism 5 and the dosages of 1/2 and 1/4 IC_50_ were used for the next studies in vitro.

Then, the HepG2-HBx cells were treated with CP at the concentration of 1/2 IC_50_ and 1/4 IC_50_ to observe the effect of CP on HepG2-HBx cells proliferation. Cyclopamine (70*μ*M), SHH pathway inhibitor, was used in our studies.

### 2.4. Migration Assay

HepG2-HBx cells could be scratched at interval 0.5cm in 6-well plate and were treated with CP (1/2 IC_50_; 1/4 IC_50_, respectively) and cyclopamine (70*μ*M) for 48 h, respectively. HepG2-NC cells were treated without any medicine. The cells could be pictured by Fluorescence Inversion microscope system (OLYMPUS, USA).

### 2.5. Clone Formation

3 × 10^2^ HepG2-HBx cells and HepG2-NC cells were cultured in 6-well plate for 2 days. On 3 day, the HepG2-HBx cells were treated with cyclopamine (70*μ*M) and CP (1/2 IC_50_; 1/4 IC_50_, respectively). After 14 days, the cells were fixed with 4% paraformaldehyde and stained with Giemsa (Leagene, Beijing). The pictures were performed by Single Lens Reflex (Canon, Japan).

### 2.6. Real-Time PCR

Total RNA was isolated by using TRIzol reagent (Takara, Japan). The first-strand cDNA in a 10uL volume was carried out with conditions comprised 15 min at 30°C, 42°C for 60 min, and 72°C for 10 min. Real-time PCR was carried out using the SYBR Premix Ex Taq (Takara, Japan) according to the manufacturer's protocol with ABI-7500 (ABI, USA). Briefly, each PCR was measured in duplicate in a 20uL volume cycling conditions comprised 2 min at 50°C, followed by 40 cycles at 95°C for 10 min, 95°C for 15s, 60°C for 1 min, 95°C for 30s, and 60°C for 15 min. *β*-actin was used as the internal control. The mRNA relative expression of HBx, SHH, PTCH-1, SMO, GLI-1, and GLI-2 was quantified using 2^−ΔΔCT^ method. Primer sequences are shown in Table 1.

### 2.7. Western Blot Analysis

Total protein was extracted from the cells into the RIPA buffer (Beyotimeg, China) and supplemented with protease inhibitor PMSF (Beyotime, China). Western blot were performed by SDS-PAGE gel and transferred to a polyvinylidene fluoride (PVDF) membrane. The PVDF membranes were probed with primary antibodies: HBx, SHH, PTCH-1, SMO, GLI-1, GLI2, and GAPDH (Abcam, USA). The results were visualized with the C-DiGit imager (Li-Cor, USA), and the levels of proteins expression were normalized by GAPDH loading control.

### 2.8. Immunohistochemistry

The tumors were fixed with 4% paraformaldehyde and embedded in paraffin. The sections (5*μ*m) were incubated with antibodies: HBx, SHH, PTCH-1, SMO, and GLI-1 (Abcam, USA). The pictures were captured by Fluorescence Inversion microscope system (Olympus, USA)

### 2.9. Immunofluorescence Staining

6 × 10^4^ cells were cultured in laser confocal dishes (NEST, China). After treated for 48 h with cyclopamine and CP (1/2 IC_50_; 1/4 IC_50_, respectively), the cells were fixed by 4% paraformaldehyde and then permeabilizated followed by probed with GLI-1 and DAPI. The pictures were captured by Confocal Laser Scanning Microscope (Leica, Germany).

### 2.10. Animals and Experimental Design

All BABL/c nude mice (n=30) were purchased from Medical Experiment Center of Guangdong Province (Guangzhou, China) and were received humane care and used in accordance with the Guangzhou University Chinese Medical Animal Care and Welfare Committee. After one week of acclimatization, a total of 1 × 10^7^ HepG2-HBx cells were suspended in PBS and injected subcutaneously into the mice to build inducting HepG2-HBx cells transplant tumor models. The mice which received HepG2-HBx cells were divided into four groups (n=6 in each group): HepG2-HBx group [PBS, intragastrically (i.g.)], cyclopamine group 30 mg/kg, i.g.), and CP groups (625mg/kg and 300 mg/kg, i.g., respectively). The HepG2-NC cells were injected subcutaneously into the mice in the group of HepG2-NC to build inducting HepG2-NC cells transplant tumor models (n=6). After two weeks, the medicines were given to the mice receiving the HepG2-HBx cells when the tumors volume is 3 × 3mm^2^. Tumors size and mice weight were measured over a three-day interval. After 17 days, the mice were sacrificed, and the tumors were dissected.

### 2.11. Statistical Analysis

All parameters in our studies were expressed with mean ± standard deviation. Statistical analysis was using version 18.0 (SPSS, USA). Groups differences were performed by the Analysis of variance (ANOVA) followed by a least significant difference test (LSD).* P<0.05* was considered to be signification. Graphs were plotted by Graphpad prism version 5 (GraphPad Software, San Diego, CA, USA).

## 3. Results

### 3.1. HPLC Analysis of CP

**Figure 1:**
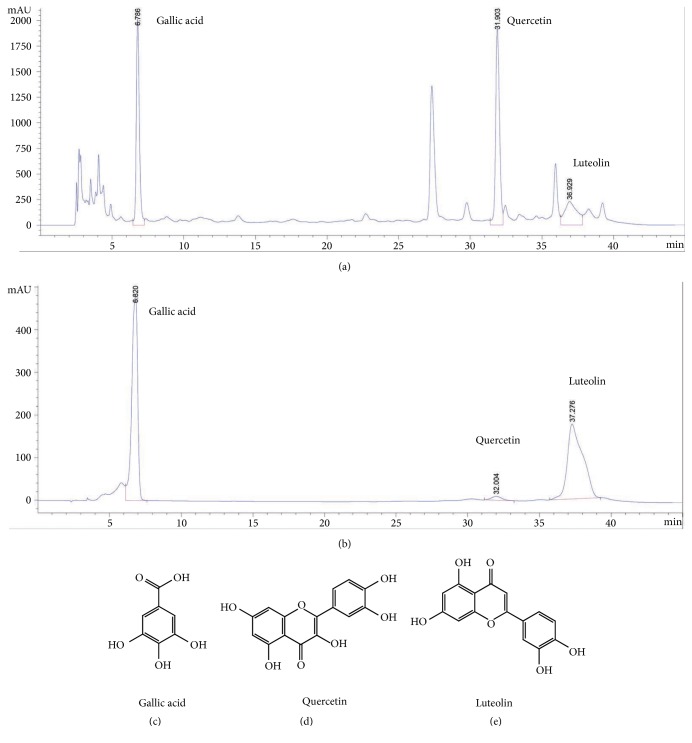
Representative high-performance liquid chromatography profile of CP. Gallic acid, quercetin, and luteolin were detected at 270 nm. (a) and (b) represented HPLC analysis of CP and standard compounds, respectively; (c), (d), and (e) presented the structures of gallic acid, quercetin, and luteolin, respectively.

The ingredients of CP and standard compounds were analyzed by HPLC at 270 nm. Gallic acid, quercetin, and luteolin were detected in CP (Figure 1(a)), with their total peak area percentages were 4.2%, 8.9%, and 11.7%, respectively. The standard compounds were measured by HPLC (Figure 1(b)). The structures of gallic acid, quercetin, and luteolin were represented (Figures 1(c), 1(d), and 1(e), respectively).

### 3.2. *CP *Could Inhibit Proliferation of HepG2-HBx Cells

**Figure 2:**
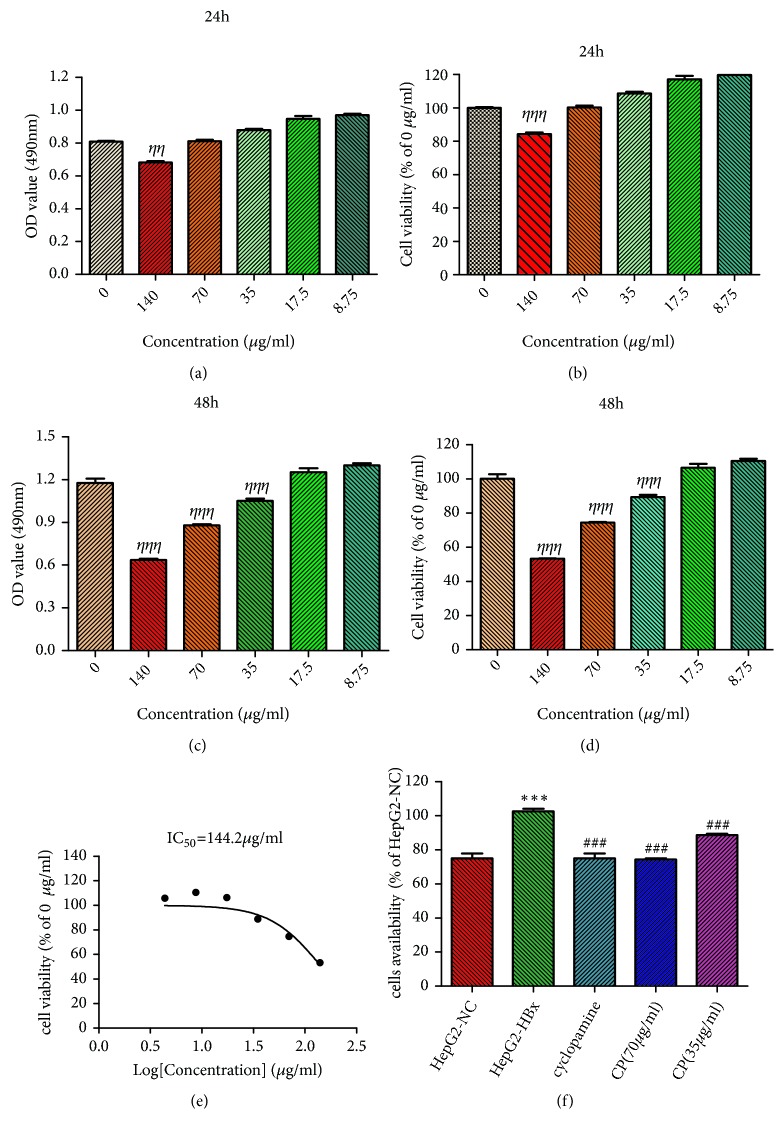
CP could inhibit proliferation of HepG2-HBx cells. Being treated for 24 h (a, b) and 48 h (c, d), the proliferation was measured by MTT. IC_50_ of the CP was calculated using the Graphpad prism soft (e). Then we measured the proliferation of HepG2-HBx cells administrated with CP or cyclopamine (70*μ*M) for 48 h (f). ^*ηηη*^*P* < 0.001, compared with HepG2-HBx cells administration with 0 *μ*g/ml. ^*∗∗∗*^*P* < 0.001, compared with the group of HepG2-NC. ^###^*P* < 0.001, compared with the group of HepG2-HBx.

To evaluate the effect of CP on the HepG2-HBx cells, we analyzed the cells proliferation using MTT. Being treated for 24 h, only the CP dosages of 140 *μ*g/ml could inhibit HepG2-HBx cells proliferation (Figures 2(a) and 2(b)). However, CP could obviously inhibit the proliferation when HepG2-HBx cells were treated for 48 h (Figures 2(c) and 2(d)). We calculated the IC_50_ of CP which is 144.2*μ*g/ml using the Graphpad prism soft when HepG2-HBx cells were treated for 48 h (Figure 2(e)). The CP dosages of 70 *μ*g/ml and 35 *μ*g/ml could be used for the next studies in vitro.

We found that HBx obviously promoted the proliferation of HepG2 cells, compared with the group of HepG2-NC. At the same time, cyclopamine (70*μ*M), a SHH pathway inhibitor, could suppress the HepG2-HBx cells proliferation compared with the group of HepG2-HBx. We also found that it could obviously decrease the proliferation of HepG2-HBx cells when HepG2-HBx cells are treated with CP (70*μ*g/ml; 35 *μ*g/ml, respectively) for 48 h, compared with the group of HepG2-HBx (Figure 2(f)).

### 3.3. *CP* Could Inhibit Clone Formation and Migration of HepG2-HBx Cells

**Figure 3:**
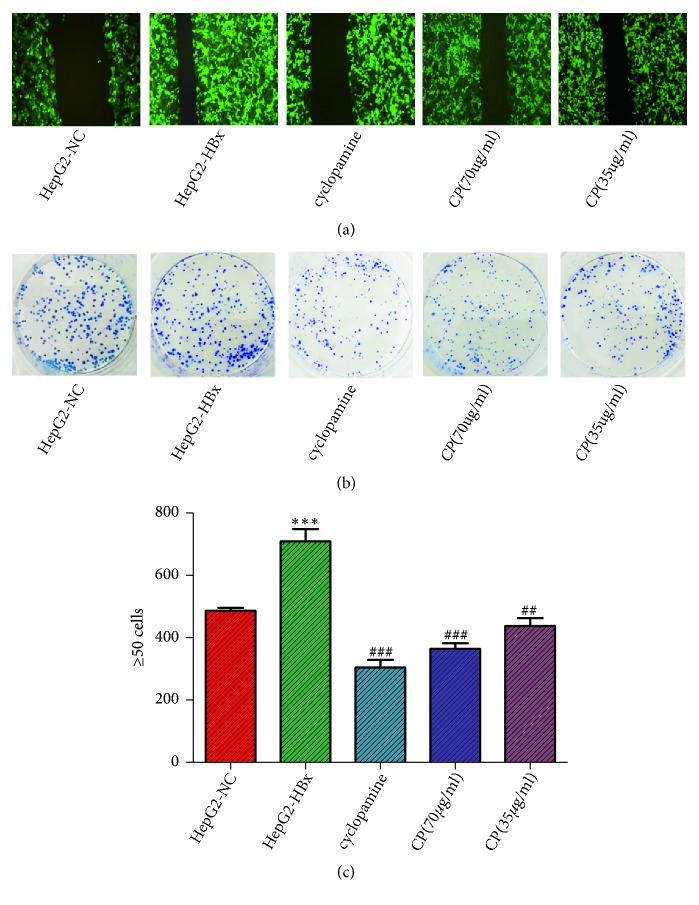
CP could inhibit migration and clone formation of HepG2-HBx cells. Migration (a), clone formation (b), and the clone numbers ≥50 cells (c) were measured when HepG2-HBx cells were treated with CP (70*μ*g/ml; 35 *μ*g/ml, respectively) and cyclopamine (70*μ*M) for 48 h. The pictures were captured by the Fluorescence Inversion microscope system (×10) or Single Lens Reflex. ^*∗∗∗*^*P* < 0.001, compared with the group of HepG2-NC; ^##^*P* < 0.01 and ^###^*P* < 0.001, compared with the group of HepG2-HBx.

Cell migration is a physiological process of normal growth and involves in embryo development, angiogenesis, wound healing, immune response, inflammation, and cancer metastasis. At the same time, migration also indicates the capability of invasive and metastasis. Oncogenic HBx promoted HepG2 cell migration, whereas the migration in HepG2-HBx cell administration CP (70*μ*g/ml; 35 *μ*g/ml, respectively) was reduced compared with the group of HepG2-HBx (Figure 3(a)).

Clone formation is an indicator of cells proliferation rate and a capability of adaptability to living environment. HBx could accelerate the clone formation of HepG-2 cells compared with HepG2-NC cells. CP could inhibit the clone formation in HepG2-HBx cells depending on does-effect relationship (Figure 3(b)). And we also measured the clone numbers*⩾*50 cells. As expected, HBx significantly increased the clone numbers*⩾*50 cells compared with the HepG2-NC group; the clone numbers*⩾*50 cells were decreased in HepG2-HBx cells treatment with CP (70*μ*g/ml; 35 *μ*g/ml, respectively) (Figure 3(c)). Collectively, all of the data we presented indicated that HBx could robustly increase the proliferation, migration, and clone formation, whereas CP could significantly inhibit the proliferation, migration, and clone formation of HepG2-HBx cells in vitro.

### 3.4. *CP* Decreased the mRNA Expressions of HBx, SHH, PTCH-1, and GLI In Vitro

**Figure 4:**
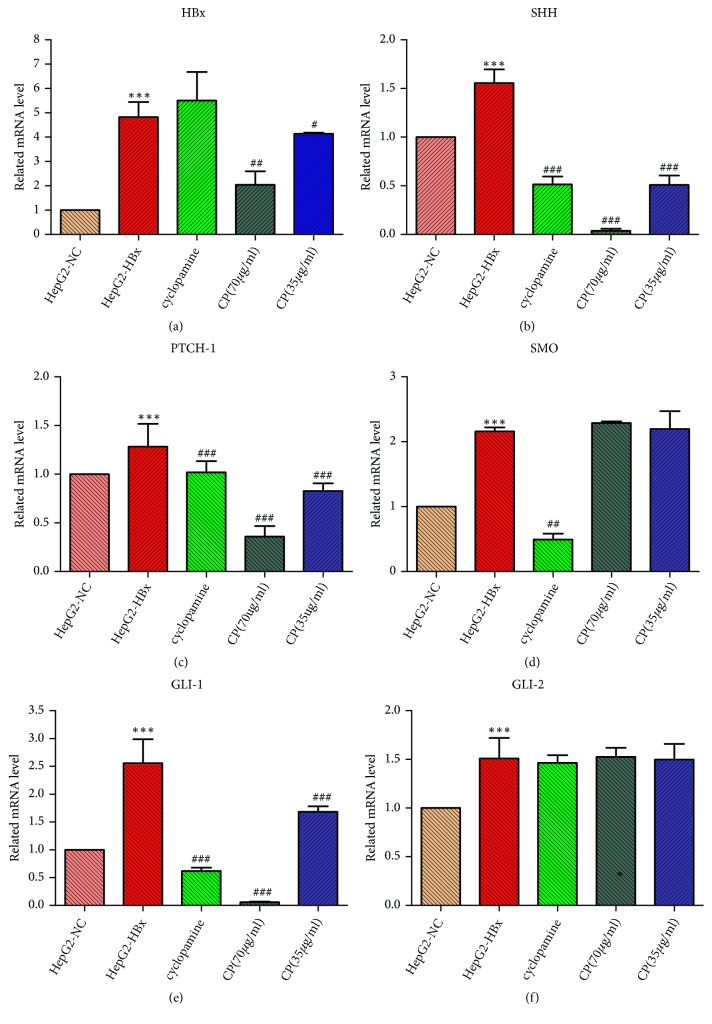
CP decreased the mRNA expressions of HBx, SHH, PTCH-1, and GLI-1 in HepG2-HBx cells. The mRNA was measured when HepG2-HBx cells were treated with CP (70*μ*g/ml; 35 *μ*g/ml, respectively) and cyclopamine (70*μ*M)for 48 h using RT-PCR. ^*∗∗∗*^*P* < 0.001, compared with the group of HepG2-NC; ^#^*P* < 0.05, ^##^*P* < 0.01, and ^###^*P* < 0.001, compared with the group of HepG2-HBx.

Based on our previous studies, we predicted that CP which inhibited HBV-related HCC might be through HBx-SHH pathway axis inactivation. To validate our prediction, we measured the mRNA expressions of HBx, SHH, PTCH-1, SMO, GLI-1, and GLI-2 in vitro. Our data revealed that mRNA expression levels of SHH, PTCH-1, SMO, GLI-1, and GLI-2 were robustly elevated in HepG2-HBx cells compared with the HepG2-NC cells, which showed that HBx could activate the SHH pathway in HepG2 cells. As we expected, the mRNA expressions of SHH, PTCH-1, and GLI-1 were significantly decreased in HepG2-HBx cells treatment with CP (70*μ*g/ml; 35 *μ*g/ml, respectively). What is more, CP not only represses the above factors, but also could inhibit HBx in a dose-effect relationship compared with the group of HepG2-HBx. However, CP (70*μ*g/ml; 35 *μ*g/ml, respectively) has no inhibition on SMO and GLI-2 (Figure 4).

### 3.5. *CP* Inhibited the Proteins Expression of HBx, SHH, PTCH-1, and GLI-1 In Vitro

**Figure 5:**
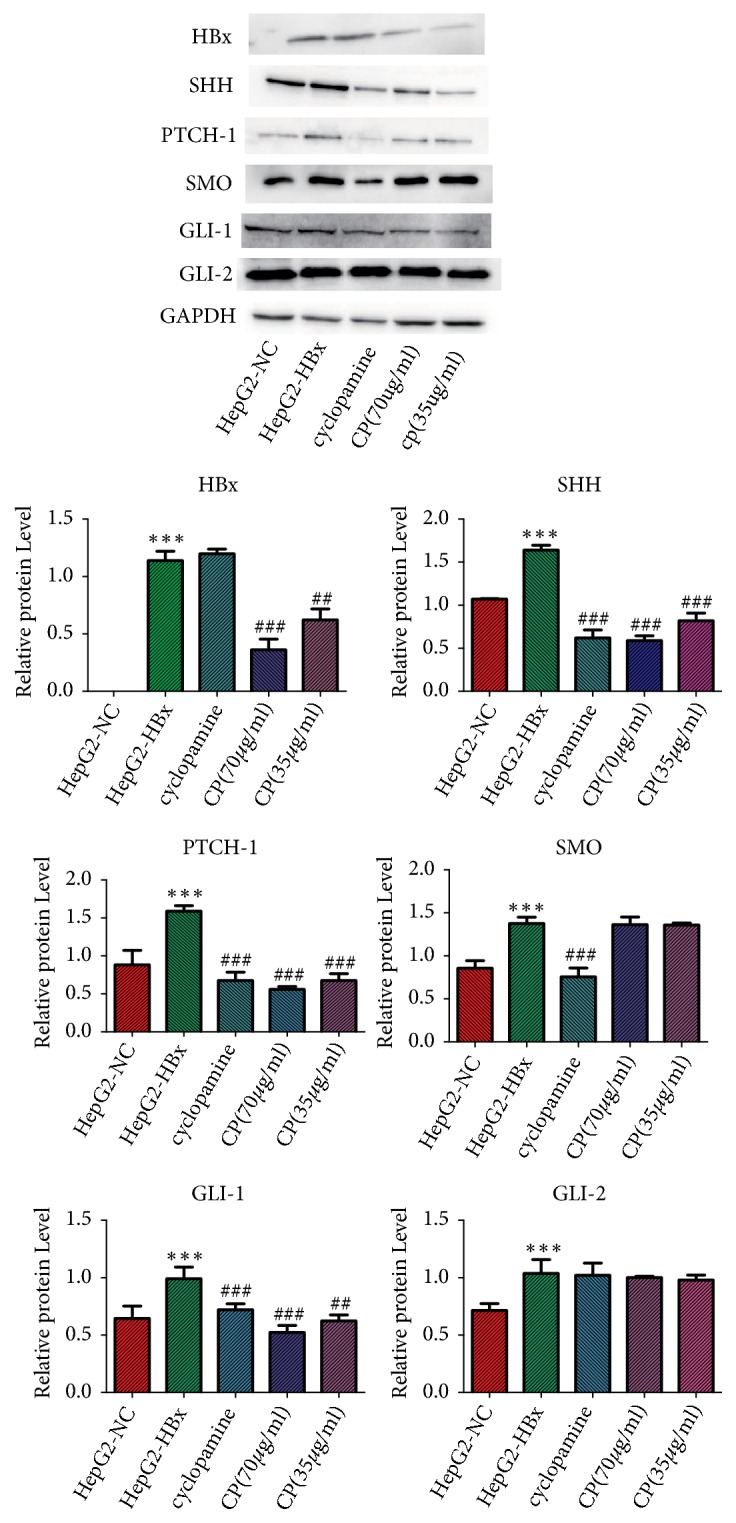
CP decreased the proteins expressions of HBx, SHH, PTCH-1, and GLI-1 in HepG2-HBx cells. HepG2-HBx cells were treated with CP (70*μ*g/ml; 35 *μ*g/ml, respectively) and cyclopamine (70*μ*M) for 48 h. Then the proteins expressions were measured by Western bloat. The related protein expressions were standard by GAPDH. ^*∗∗∗*^*P* < 0.001, compared with the group of HepG2-NC; ^##^*P* < 0.01 and ^###^*P* < 0.001, compared with the group of HepG2-HBx.

To further elucidate the speculation, we measured the proteins expression in HepG2-HBx cells treatment with CP. In accordance with the results of mRNA, HBx could increase proteins expression of SHH, PTCH-1, SMO, GLI-1, and GLI-2 in HepG2-HBx cells compared with the HepG2-NC cells, which implied that HBx could activate the SHH pathway in HepG2 cells. The proteins expression of SHH, PTCH-I, and GLI-1 was downregulated in HepG2-HBx cells treatment with CP (70*μ*g/ml; 35 *μ*g/ml, respectively) compared with HepG2-HBx cells. CP not only inhibited the proteins expression of SHH, PTCH-1, and GLI-1, but also could reduce the protein expression of HBx, compared with the HepG2-HBx cells. However, CP (70*μ*g/ml; 35 *μ*g/ml, respectively) could not reduce the proteins expression of SMO or GLI-2 (Figure 5). Taken together, all the results of mRNA and proteins implied that HBx activated the SHH pathway in HepG2 cells, and CP could inactivate HBx, SHH, PTCH-1, and GLI-1 in HepG2-HBx cells.

### 3.6. CP Could Inhibit the GLI-1 Expression in Cytoplasm

**Figure 6:**
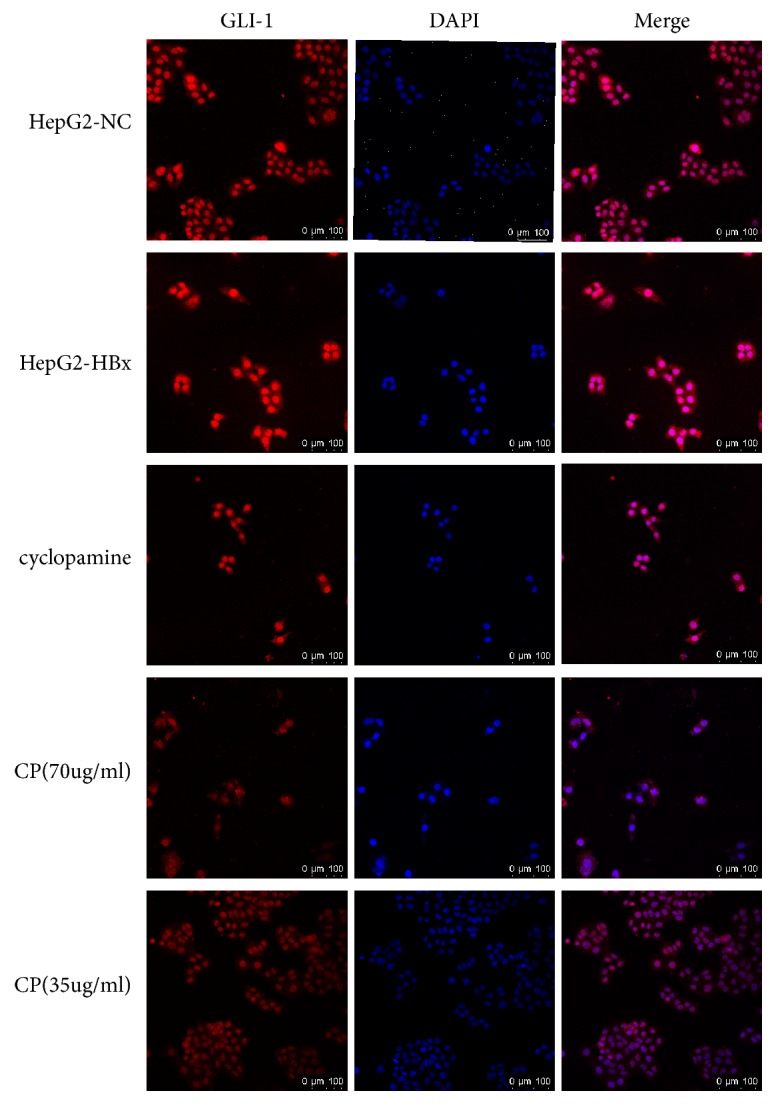
CP could inhibit the GLI-1 expression in cytoplasm. The expression of GLI-1 in HepG2-HBx cells treated with CP (70*μ*g/ml; 35 *μ*g/ml, respectively) and cyclopamine (70*μ*M) was measured by immumofluorescence. And the pictures were captured by Confocal Laser Scanning Microscope.

GLI-1 is an indicator of the SHH pathway activation. From the data from the mRNA and protein, we found that the HBx could increase the expression of GLI-1 in HepG2-HBx cells. In order to clearly observe the change of GLI-1 in HepG2-HBx cells treated with CP (70*μ*g/ml; 35 *μ*g/ml, respectively), we measured the level of GLI-1 in cytoplasm by immumofluorescence. As expected, HBx could augment fluorescence intensity of GLI-1 in cytoplasm compared with the HepG2-NC cells. And CP (70*μ*g/ml; 35 *μ*g/ml, respectively) could robustly attenuate the expression of GLI-1 in cytoplasm compared with the HepG2-HBx cells (Figure 6). Our data showed that CP could reduce the expression of GLI-1 in cytoplasm.

### 3.7. CP Could Inhibit Tumors Growth in Mice

**Figure 7:**
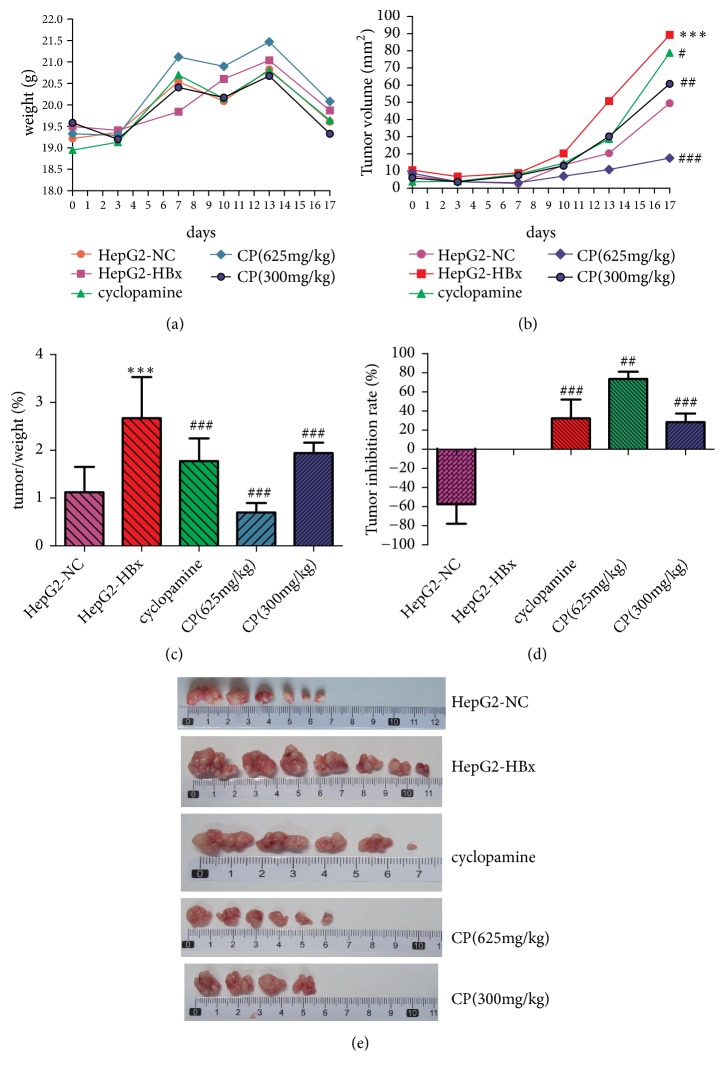
CP could inhibit tumors growth in vivo. We transplanted the HepG2-NC cells and HepG2-HBx cells into Babl/c nude mice, respectively. Body weights (a) and tumor volume (b, e) were measured at the indicated times. We measured the tumor/weight (%) (c) and tumor inhibition rate (%) (d) at the end of experiment. ^*∗∗∗*^*P* < 0.001, compared with the group of HepG2-NC; ^#^*P* < 0.05, ^##^*P* < 0.01, and ^###^*P* < 0.001, compared with the group of HepG2-HBx.

To validate our results in vivo, we transplanted the HepG2-NC cells and HepG2-HBx cells into the Babl/c nude mice to observe the effect of CP on HBV-related HCC. In our study, the mice weight of CP (625mg/kg) was heavier than the group of HepG2-NC and HepG2-HBx, but no statistic significance (Figure 7(a)). Oncogene HBx increased the tumor volume and tumor/weight (%) in mice of HepG2-HBx group compared with that of HepG2-NC group, which showed that HBx could promote the tumor growth. The tumor volume and tumor/weight (%) were significantly decreased in CP (625mg/kg and 300 mg/kg) groups compared with the group of HepG2-HBx (Figures 7(b), 7(c), and 7(e)). HBx could decrease the tumor inhibition rate in HepG2 cells, which implied that HBx promoted the tumor growth. CP (625mg/kg; 300 mg/kg) could elevate the tumor inhibition rate (%) compared with HepG2-HBx group (Figure 7(d)). All of our data showed that HBx promoted tumor growth and CP (625mg/kg and 300 mg/kg) could inhibit growth of tumor in dose-effect relationship.

### 3.8. CP Could Inhibit the mRNA Expression of HBx, SHH, PTCH-1, and GLI-1 In Vivo

**Figure 8:**
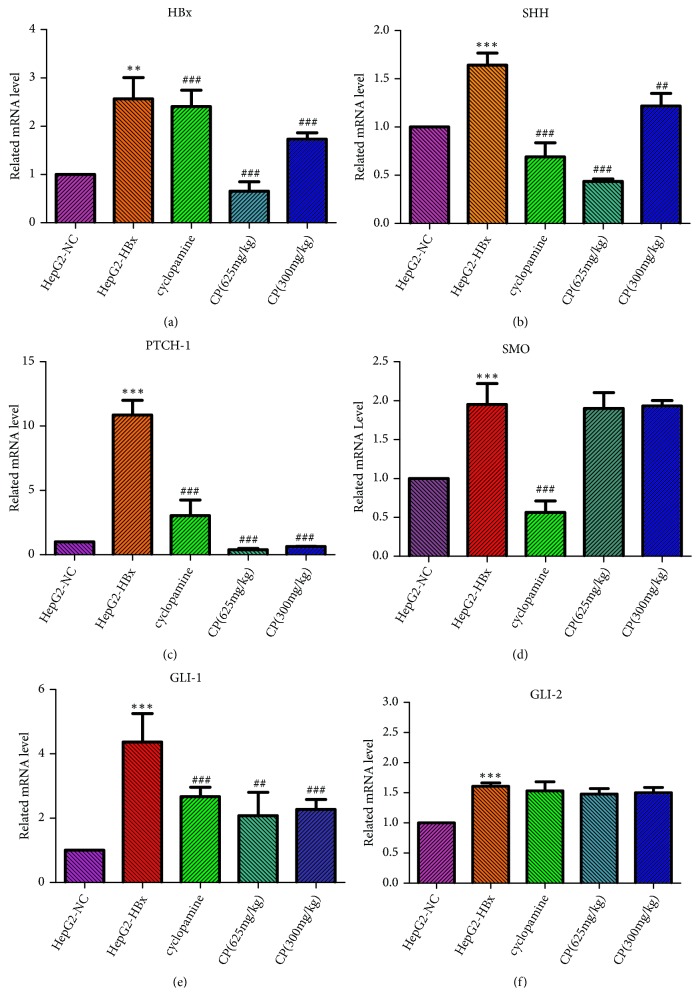
CP could inhibit the mRNA expression of SHH, PTCH-1, GLI-1, and HBx in vivo. The mRNA levels of HBx (a), SHH (b), PTCH-1 (c), SMO (d), GLI-1 (e), and GLI-2 (f) in tumors were measured by RT-PCR. ^*∗∗*^*P* < 0.01 and ^*∗∗∗*^*P* < 0.001, compared with the group of HepG2-NC. ^##^*P* < 0.01 and ^###^*P* < 0.001, compared with the group of HepG2-HBx.

We had verified that CP could inactivate the HBx, SHH, PTCH-1, and GLI-1 in vitro. The data from in vivo also demonstrated that CP could inhibit the tumors growth. In order to further confirm our assumption, we observed the expressions of mRNA in tumors. As same as the data from in vitro, CP (625mg/kg; 300 mg/kg) could reduce the mRNA expressions of HBx, SHH, PTCH-1, and GLI-1 in dose-effect relationship compared with the group of HepG2-HBx. However, CP could not inhibit the mRNA expressions of SMO and GLI-2 (Figure 8).

### 3.9. CP Could Repress the Proteins Expression of HBx, SHH, PTCH-1, and GLI-1 In Vivo

**Figure 9:**
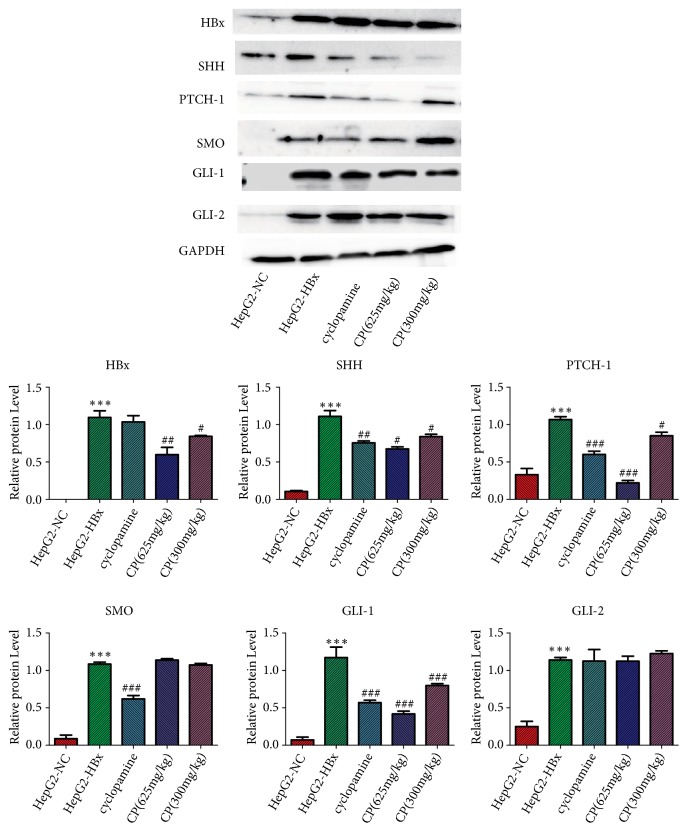
CP could inhibit the proteins expression of HBx, SHH, PTCH-1, and GLI-1 in tumors. We measured the proteins level of HBx, SHH, PTCH-1, SMO, GLI-1, and GLI-2 in vivo. ^*∗∗∗*^*P* < 0.001, compared with the group of HepG2-NC; ^#^*P* < 0.05, ^##^*P* < 0.01, and ^###^*P* < 0.001, compared with the group of HepG2-HBx.

We observed the proteins expression of HBx, SHH, PTCH-1, SMO, GLI-1, and GLI-2 in vivo. Being consistent with the mRNA, the proteins expression of SHH, PTCH-1, SMO, GLI-1, and GLI-2 was significantly increased in the group of HepG2-HBx compared with the group of HepG2-NC. CP with two dosages (625mg/kg and 300 mg/kg, respectively) could reduce proteins levels of HBx, SHH, PTCH-1, and GLI-1 in a dose-dependent manner. However, CP could not inhibit the proteins expression of SMO and GLI-2 (Figure 9).

### 3.10. CP Could Inactivate the SHH Pathway in Tumors

**Figure 10:**
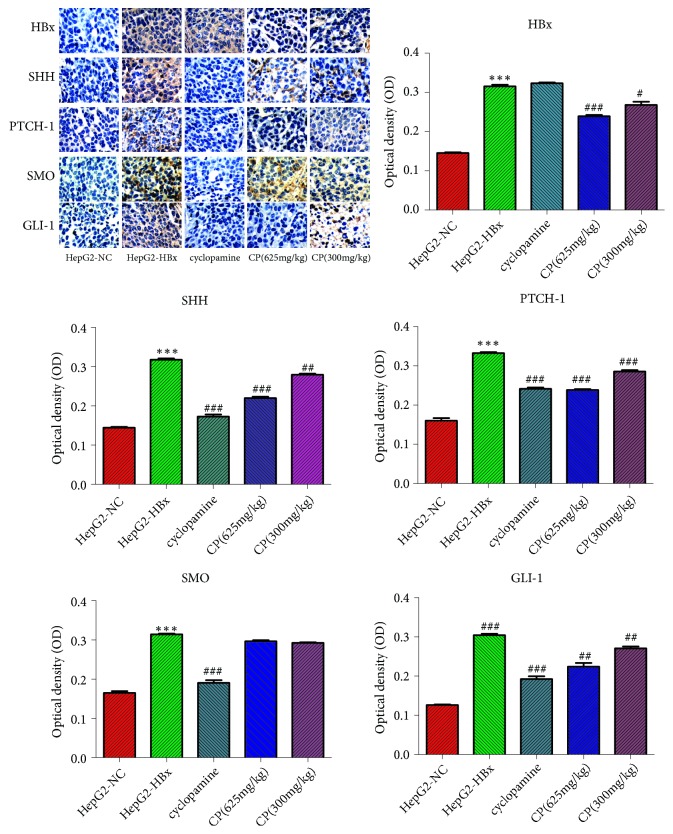
CP could inactivate the SHH pathway in tumors. We measured the expression levels of HBx, SHH, PTCH-1, SMO, and GLI-1 in tumors with immunohistochemistry. ^*∗∗∗*^*P* < 0.001, compared with the group of HepG2-NC; ^##^*P* < 0.01 and ^###^*P* < 0.001, compared with the group of HepG2-HBx.

We observed the expression of HBx, SHH, PTCH-1, and GLI-1in tumors with Immunohistochemistry. The results were consistent with the expressions of mRNA and protein. HBx could increase the expression of SHH, PTCH-1, SMO, and GLI-1 in the group of HepG2-HBx compared with the group of HepG2-NC. The mice treatment with CP (625mg/kg and 300 mg/kg) could decrease the expression of HBx, SHH, PTCH-1, and GLI-1 in a dose-dependent manner. However, CP could not inhibit the protein expression of SMO in tumors (Figure 10).

## 4. Discussion

Here, we investigated the effects of CP on HBV-related HCC in vitro and in vivo. All of the data we presented revealed that HBx activate the SHH pathway to promote the HBV-related HCC development. And CP has significant anti-HBV-related HCC activity that might be mediated by inactivation of HBx, SHH, PTCH-1, and GLI-1.

CP is made up of* Phyllanthus urinaria L, Astragalus membranaceus (Fisch.) Bunge, Scutellaria barbata D. Don, Curcuma zedoaria (Christm.) Rosc, and Cremastra appendiculata (D. Don) Makino.* It is a formula used for HCC, especially HBV-related HCC in China. In our previous studies, we have elucidated that CP could reduce the expression of HBxAg in transplant tumors, and we also verified that CP could inhibit HBV-DNA replication and reduce the expression of URG4, URG7, URG11, and URG9, preventing and delaying the development of HBV-related HCC in patients treated with CP for two years [16–18]. Other scholars proved that CP could inhibit expressions of HBsAg and HBeAg, and it also could enhance the immune response in vivo and in vitro [19].* Phyllanthus urinaria L* extract could reduce the expression of HBx and also could deregulate miR-21 and upregulate miR-145 [14, 20]. As the constituents of CP, gallic acid, quercetin, and luteolin have anticancer activities. Quercetin can suppress miR-21 to alleviate TGF-*β*-induced fibrosis and activate the JNK pathway to induce apoptosis in KRAS-mutant colorectal cancer cells and also could inhibit the SHH pathway to regulate pancreatic cancer stem cell characteristics [21]. Gallic acid could inhibit the proliferation and induce apoptosis in HepG2 and SMMC-7721 cells via mitochondrial-mediated pathway, and it also can reduce the replication of HCV [22]. Luteolin could promote cells apoptosis by inducing autophagy in SMMC-7721 cells [23].

Although CP and its constituents present the capability of anticancer, the molecular mechanism of CP on HBV-related HCC remains not fully uncovered. HBx, as an oncogene, plays a vital role in pathogenesis and tumorigenesis in HBV-related HCC. Cross-talking with hypoxia-inducible factor-1 alpha (HIF-1alpha) via MAPK pathway, HBx leads to activate of HIf-1 alpha target genes, which contribute to HBV-related HCC development [24]. And HBx also can interact with absent in melanoma 2 (AIM2) to lead to HCC metastasis [25]. And* Kim et al.* confirmed that HBx could stimulate Hedgehog-Gli pathway activation to promote the tumorigenesis [26]. At the same time, we found that HBx has a positive correlation with the SHH pathway in liver tissues of HBV-related HCC patients. Several lines of evidence confirm that abnormality of the SHH pathway plays an important role in the liver diseases.* Chen et al.* validated that deregulating the GLI-1 could inhibit the migration and invasion in hepatocellular carcinoma.* Kim et al.* have found that hepatic stellate cells could stimulate the proliferation and migration of cholangiocarcinoma cells through HH signaling activation [27]. As the SHH pathway inhibitor, cyclopamine could induce cell apoptosis by downregulating BCL-2 in HCC [28].

Based on our previous studies, we predicted that CP has an anti-HBV-related HCC capability, which might be regulated by inactivation of HBx-SHH pathway axis. As expected, in our studies, all of data in vivo and in vitro further confirmed that HBx could increase the expression of the SHH pathway compositions: SHH, PTCH-1, SMO, GLI-1, and GLI-2, contributing to HCC development. More importantly, CP not only reduced the expression of HBx, but also could inactivate the SHH, PTCH-1, and GLI-1 to inhibit the tumors growth. However, CP could not decrease the expression of SMO and GLI-2.

Notably, from proteins analysis (Figures 5 and 9) and immunofluorescence staining (Figure 6), we found that the SHH pathway components were also detected in HepG2-NC cells, which implied that maybe the SHH pathway also was activated in naïve HepG2 cells. So, it is worthwhile to explore the effect of CP on naïve HepG2 cells, which would confirm whether there is effect of CP on SHH pathway just through the inhibition of HBx or not.

In conclusion, our data presented here indicated that CP could repress expression of HBx and inactivate the SHH pathway in a dose-dependent manner, which might be one of underlying mechanisms of CP anti-HBV-related HCC.

## Figures and Tables

**Table 1:** Primer sequences used in RT-PCR.

Genes	Primer sequence	
GLI-1	Forward	CTGGATCGGATAGGTGGTCT
	Reverse	CAGAGGTTGGGAGGTAAGGA
GLI-2	Forward	GCCCTTCCTGAAAAGAAGAC
	Reverse	CATTGGAGAAACAGGATTGG
SHH	Forward	AAAGCTGACCCCTTTAGCCTA
	Reverse	TTCGGAGTTTCTTGTGATCTTCC
PTCH-1	Forward	CCGTTCAGCTCCGCACAGA
	Reverse	CTCACTCGGGTGGTCCCATAAA
SMO	Forward	GAGCGTAGCTTCCGGGACTA
	Reverse	CTGGGCCGATTCTTGATCTCA
HBx	Forward	TTTTCAGAGGCCAGTCAACGA
	Reverse	GAGCCCTGTCAGGTCCACAA
*β*-ACTIN	Forward	ATCGTGCGTGACATTAAGGAGAAG
	Reverse	AGGAAGGAAGGCTGGAAGAGTG

## Data Availability

The data used to support the findings of this study are available from the corresponding author upon request.
